# Enjoyment of life predicts reduced type 2 diabetes incidence over 12 years of follow-up: findings from the English Longitudinal Study of Ageing

**DOI:** 10.1136/jech-2020-214302

**Published:** 2020-10-21

**Authors:** Laura Panagi, Ruth A Hackett, Andrew Steptoe, Lydia Poole

**Affiliations:** 1 University College London, London, UK; 2 King’s College London, London, UK

**Keywords:** Psychosocial factors, Diabetes, Longitudinal studies, Epidemiology

## Abstract

**Background:**

Subjective well-being appears to be associated with reduced risk of type 2 diabetes (T2D). However, it is unknown whether this association is similar across different types of well-being. We examined the relationship between hedonic and eudaimonic well-being and incident T2D, and explored the role of sociodemographic, behavioural and clinical factors in these associations.

**Methods:**

We used data from 4134 diabetes-free participants from the English Longitudinal Study of Ageing (mean age =64.97). Enjoyment of life and purpose in life were assessed using items from the CASP-19 to reflect hedonic and eudaimonic well-being, respectively. Participants reported T2D diagnosis over 12 years. We used Cox proportional hazards regression analyses and also explored the percentage of association explained by different covariates.

**Results:**

Results revealed a protective role for enjoyment of life in T2D rate adjusting for sociodemographic (age, sex, wealth, ethnicity, marital status), behavioural (physical activity, smoking, alcohol consumption, body mass index) and clinical (hypertension, coronary heart disease and glycated haemoglobin) characteristics (HR =0.93, p=0.021, 95% CI (0.87, 0.99)). Sociodemographic, behavioural and clinical factors accounted for 27%, 27% and 18% of the association, respectively. The relationship between purpose in life and T2D was non-significant (adjusted HR =0.92, p=0.288, 95% CI (0.78, 1.08)).

**Conclusion:**

This study illustrates how the link between subjective well-being and T2D varies between well-being components. It also demonstrates that sociodemographic, behavioural and clinical factors partially explain this association. Intervention studies examining whether changes in enjoyment of life can help delay T2D onset are warranted.

## INTRODUCTION

Subjective well-being is a multidimensional concept that is often divided into two subcomponents: hedonic and eudaimonic well-being. Hedonic well-being encompasses positive feelings such as enjoyment of life, joy and happiness. Eudaimonic well-being refers to judgements about sense of purpose in life and self-realisation^1^. Compelling evidence from longitudinal studies demonstrates the health-protective role of hedonic and eudaimonic well-being among older adults,^2 3^ including those with type 2 diabetes (T2D^4^).

T2D is a progressive, metabolic disorder that is highly prevalent among the older population. Growing evidence suggests a protecting role for subjective well-being in T2D. One previous study using data from the English Longitudinal Study of Ageing (ELSA), a nationally representative study of adults >50 years old living in England, found an independent, inverse relationship between an aggregate measure of well-being and T2D incidence,^5^ but only in participants younger than 65 years. A more recent study including a national sample of 3907 older adults examined the association between purpose in life and incident pre-diabetes or T2D over four years of follow-up. Results showed that participants with a higher sense of purpose in life at baseline had lower risk of developing pre-diabetes or T2D (as indexed by glycated haemoglobin (HbA1c) measurements) after adjusting for demographic variables, physical health and physical function at baseline, depression and psychiatric diagnoses.^6^ In another study with 7800 participants, emotional vitality but not optimism was associated with a 9–15% decrease in the odds of having self-reported doctor-diagnosed T2D 13 years later.^7^ Results remained significant after adjusting for demographic, lifestyle and clinical risk factors but were attenuated when depressive symptoms were included in the model. In participants with a parental history of diabetes, positive emotions were associated with a lower risk of diabetes after controlling for sociodemographic and health measures as well as negative and depressed affect.^8^

Although the different aspects of well-being are moderately positively inter-correlated, it is recognised that enjoyment of life may not be always accompanied by a sense that life is worthwhile, and vice versa.^9^ The strength of the associations with health outcomes may also vary across types of well-being.^7^ For example, optimism has emerged as a robust predictor of cardiovascular health, although associations are less consistent for cancer.^10^ It has therefore been argued that consideration of the individual contribution of the different types of well-being is worthwhile in health research.^9^

Previous studies have demonstrated an independent association between subjective well-being and future T2D. Nevertheless, the extent to which this relationship can be explained by other factors remains unclear. Sociodemographic characteristics, including wealth and socioeconomic position, are associated with diabetes onset and progression with findings supporting a social gradient in health.^11^ Health behaviours present another plausible pathway through which well-being might affect T2D risk. Regular physical activity, for example, is consistently associated with hedonic well-being, and its effect on glucose metabolism and body weight at older ages has been well established.^12^ Other health behaviours such as smoking and alcohol consumption are also relevant both to reduced hedonic well-being, ^13^ ^14^ and diabetes risk.^15 16^ Also, hedonic and eudaimonic well-being are associated with a more favourable cardiovascular prolife in healthy and diseased populations,^17^ which in turn can reduce the risk of T2D.^18^

We aimed to test the separate effect of enjoyment of life and purpose in life on incident T2D, and to estimate the amount of association explained by sociodemographic, lifestyle and clinical factors. It was hypothesised that higher enjoyment of life and a stronger sense of purpose in life would be associated with a reduced rate of T2D onset, and that these effects would be, at least in part, explained by sociodemographic, behavioural and clinical characteristics. Considering the findings from previous research which indicated that the effect of hedonic well-being is not secondary to the absence of depressed or negative affect,^5 8 19^ we also hypothesised that these relationships would be robust to adjustment for depression. Finally, given the age- and sex-dependent findings reported in some of previous studies of different concepts of well-being and T2D,^5 19 20^ we further explored whether age or sex moderate associations between subjective well-being and T2D.

## METHODS

### Participants

The current study used data from ELSA, a panel study of men and women aged 50 years and older living in England. Since the first data collection phase in 2002–2003 (referred to as Wave 1), the sample has been followed-up biennially.^21^ Self-reported questionnaires and personal interviews are carried out at each wave and nurse visits are conducted at alternate waves for objective biological, physical and anthropometric measurements. ELSA was approved by the London Multicentre Research and Ethics Committee (MREC/01/02/91) and all participants provided fully informed written consent.^21^

In the present study, we tested associations between two different domains of subjective well-being measured at Wave 2 (2004/2005) and incident T2D from Wave 3 (2006/2007) through to Wave 8 (2016/2017). The first HbA1c data were collected at Wave 2 (2004/2005); thus, Wave 2 was selected as the baseline. A total of 8780 members participated at baseline. Wave 8 (2016/2017) is the most recently completed phase of data collection that is available for analysis. Participants in the current study were followed-up for 11.6 years on average.

**Figure 1:**
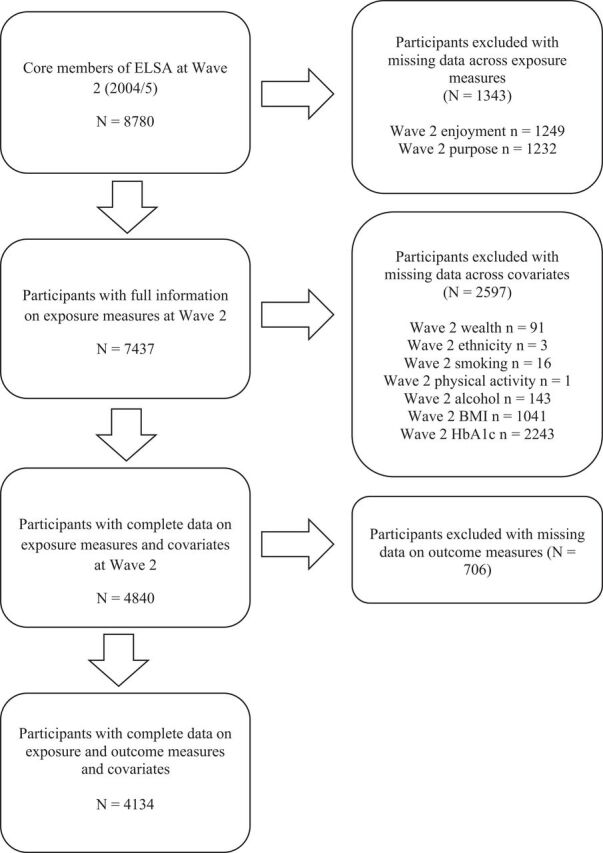
Flow diagram of those included and excluded from the analysis. ‘n’ represents the number of missing data on each variable; some participants had missing data on more than one variable. BMI, body mass index; CHD, coronary heart disease; ELSA, English Longitudinal Study of Ageing; HbA1c, glycated haemoglobin; N, number.

Participants were free from self-reported diabetes or high blood sugar diagnosis at baseline (2004/2005). Participants were excluded from the analysis if they had incomplete data (one or more missing) on exposure measures (n=1343) or any of the covariates (n=2597). Participants were included in analysis if they provided follow-up data on diabetes incidence on at least one wave. Therefore, 706 participants with missing outcome data were also excluded from the analysis. These exclusion criteria resulted in an analytical sample of 4134 participants. A flow diagram of the sample size is depicted in figure 1. Sociodemographic, behavioural and clinical characteristics were compared between the analytical sample (n=4134) and those excluded due to missing data (n=4646). Significant differences were observed in age, financial wealth, ethnicity, marital/cohabitation status, body mass index (BMI), physical activity, smoking, alcohol consumption (p=0.002), hypertension, coronary heart disease (CHD) and HbA1c (all other ps <0.001). Specifically, compared with those excluded due to missing data, included participants were younger and wealthier on average. They were more likely to be of white ethnicity and to be married or cohabiting. Additionally, participants included in the study were more likely to be non-smokers and physically active, they had lower BMI and HbA1c levels at baseline, and were less likely to have hypertension or CHD. Finally, participants of the study were more likely to consume alcohol more often that those excluded from the analysis.

### Measures

Exposure variables: enjoyment of life and purpose in life.

Enjoyment of life and purpose in life at Wave 2 (2004/2005) were assessed using items from the Control Autonomy self-realisation and Pleasure (CASP)-19 scale as measures of hedonic and eudaimonic well-being, respectively. The CASP-19 is a self-reported questionnaire which was developed to measure overall quality of life in old age.^22^ Enjoyment of life was indexed with four items from the CASP-19 (‘I enjoy the things that I do’; ‘I enjoy being in the company of others’; ‘On balance, I look back on my life with a sense of happiness’; ‘I feel full of energy these days’). Participants responded to each of the items on a 4-point Likert-type scale (from 1= never to 4= often). Total scores range from 4 to 16 with higher scores indicating higher enjoyment of life. The continuous score was used in this study. A three-level categorical variable was also created for graph purposes (low (score ≤12), middle (score =13–14) and high (score ≥15)). The CASP-19 enjoyment of life subscale has been used in previous studies of physical capability and all-cause mortality.^23 24^ The internal consistency (Cronbach’s alpha) of this subscale was 0.69 in our sample. Purpose in life was measured using one item from the CASP-19. Participants were asked to rate how often they feel that their life has meaning (from 1= never to 4= often). The continuous score was used with higher scores indicating a higher sense of purpose in life. A binary measure (low (score =1–2) and high (score =3–4)) was also created to be used in sensitivity analysis.

### Outcome variable: time to T2D

T2D was self-reported from Wave 3 (2006/2007) to Wave 8 (2016/2017). Specifically, participants were asked at each wave whether a physician had given them a diagnosis of diabetes or high blood sugar since their last interview. Time of diagnosis was indexed as the wave at which the participant first reported a diagnosis of diabetes or high blood sugar (the duration, in months, based on when diabetes was first reported).

### Covariates

We adjusted our analysis for a range of covariates. All covariates were measured at baseline (2004/2005). Self-reported socioeconomic position was indexed as quintiles of total financial wealth (gross financial wealth net of debt including savings and investments, the value of any home and other property (less mortgage), the value of any business assets and physical wealth such as artwork and jewellery). Ethnicity (white, non-white) and relationship status (married or cohabiting, neither married nor cohabiting) were also reported. Participants reported frequency of physical activity (light or none weekly, moderate or vigorous once a week, moderate or vigorous more than once a week), their smoking status (smoker, non-smoker) and frequency of alcohol consumption (≥5 times a week, <5 times a week). Height (cm) and weight (kg) were objectively measured by a nurse to calculate BMI (kilograms per square metre (kg/m^2^)), which was assessed using the continuous range of scores. Self-reported doctor diagnosis of hypertension was combined with objective blood pressure assessments carried out during the nurse visit. Hypertension was defined as systolic blood pressure ≥140 mm of Mercury and diastolic blood pressure ≥90 mm of Mercury^25^ to generate a binary variable (no, yes). Similarly, we generated a self-reported measure of prevalent CHD at baseline which included angina and/or myocardial infarction diagnosis by Wave 2 (no, yes). For HbA1c, blood samples were drawn during the nurse visit and analysed at the Royal Victoria Infirmary laboratory in Newcastle upon Tyne, United Kingdom. The International Federation of Clinical Chemistry units, millimoles per mole (mmol/mol), are reported throughout. The Diabetes Control and Complication Trial units, measured in percentage (%), are also provided.

Finally, depression status was used in a secondary analysis. Depressive symptoms were measured using Centre for Epidemiological Studies-Depression (CES-D) scale.^26^ The 8-item version was used for the purposes of this study. The psychometric properties of the 8-item version are comparable to the original 20-item version.^27^ Items included statements such as ‘I felt depressed’; ‘I felt everything I did was an effort’; ‘My sleep was restless’. A dichotomous response to each item resulted in a total score ranging between 0 (no symptoms) and 8 (all eight symptoms). A cut-off point of ≥4 was used to define significant depressive symptoms,^28^ and a combined variable of a self-reported doctor diagnosis of depression and/or a positive CES-D score were used to produce a binary depression measure (no, yes). Internal consistency was good in this sample (α =0.78).

### Statistical analysis

We first examined the association between our exposure variables (enjoyment of life and purpose in life) and sample characteristics using Pearson’s *r* correlations for continuous measures and independent samples t-tests and Kruskal-Wallis H tests for categorical measures, as appropriate.

We ascertained that the proportional hazards assumption was not violated by using log (-log (survival)) versus log (time) graphs. Thereafter, two Cox proportional hazards regression models were used to examine associations between the two different domains of well-being (enjoyment of life and purpose in life) and T2D incidence after adjustment for age, sex, financial wealth, ethnicity, marital/cohabitation status, physical activity, smoking status, alcohol consumption, BMI, hypertension, CHD and HbA1c. Furthermore, in order to estimate the proportion of the association explained by the different covariates, we built our models sequentially, calculating the percentage of protective association explained (PPAE) by the inclusion of different group of covariates.^29^ PPAE =1—((1—HR of E + X) * 100)/((1- HR of E) * 100) * 100 where HR = HR, E = exposure variable and X = explanatory variables being tested. Therefore, five separate models were tested for each exposure variable: (1) unadjusted model; (2) adjusted for age, sex, financial wealth, ethnicity and marital/cohabitation status; (3) adjusted for physical activity, smoking status, alcohol consumption and BMI; (4) adjusted for hypertension, CHD and HbA1c; (5) fully adjusted model. We treated our exposure variables as continuous scores where the HRs and 95% CIs (CIs) represent a one-unit increase. Time to event was measured in months from Wave 2 (2004/2005) to the time of the follow-up wave at which the participant first self-reported a diagnosis of diabetes or high blood sugar.

Depression status was added in secondary analysis to test if the relationship between subjective well-being and T2D is independent of depression. Furthermore, we examined whether there was a moderating effect of age by entering a mean-centred interaction term in our fully adjusted models. We also used an interaction term to test for a moderating effect of sex in the fully adjusted models.

Several sensitivity analyses were also carried out. First, we reran our main analysis after excluding participants who developed T2D within two years from baseline (by Wave 3; 2006/2007). Second, the main analysis was repeated after excluding participants with HbA1c ≥48 mmol/mol (equals to 6.5%) at baseline. This clinical cut-off point is applied for the diagnosis of T2D^30^ and it was used in the current study to reflect an objective measure of baseline T2D. Finally, since the purpose in life measure was positively skewed (skewness = −1.80, kurtosis =2.89), main analysis was reran using a binary variable (low (score =1–2) versus high (score 3–4)).

T2D incident cases are plotted on a graph to reflect the time to diagnosis for low (score ≤12), middle (score =13–14) and high (score ≥15) enjoyment categories according to baseline enjoyment of life score. The level of significance was set at p<0.05, though exact significance levels are reported throughout. All analyses were conducted using the Statistical Package for the Social Sciences version 25.

## RESULTS

A total of 4134 (55.8% women) free of T2D at baseline took part in this study. Participants were 64.97 years old on average (SD =8.99). Most of them were married or cohabiting (73.1%) and of white ethnicity (99.0%). The average BMI was within the overweight range (BMI =27.56 kg/m^2^, SD =4.63, min =14.87, max =56.15) and the mean HbA1c was 36.2 mmol/mol (SD =2.46; equals to 5.46%, SD =0.44). Mean enjoyment of life score was 14.23 (SD =1.75) and mean purpose in life score was 3.59 (SD =0.71) in this sample (possible ranges were 4–16 and 1–4, respectively). Cross-sectional associations between sample characteristics and our exposure measures are shown in table 1. Overall, people who scored higher in enjoyment of life and purpose in life were younger, married or cohabiting and wealthier. Moreover, they were more likely to be non-smokers and to engage in physical activity more frequently. Enjoyment of life but not purpose in life was associated with female gender. Higher enjoyment of life score was also associated with lower BMI and lower risk of hypertension. Higher scores in enjoyment of life and purpose in life were associated with lower risk of CHD, depression and lower HbA1c levels.

**Table 1:** Sample characteristics and associations with subjective well-being at 2004/2005 (N=4134)

Characteristic	n (%) or Mean±SD	Associations with enjoyment of life effect size, p value*	Associations with purpose in life effect size, p value*
CASP enjoyment of life score	14.23±1.75	–	*r=*0.62, p*<*0.001
CASP purpose in life score	3.59±0.71	*r=*0.62, p*<*0.001	*–*
Age (years)	64.97±8.99	*r*=−0.05, p*=*0.003	*r*=−0.03, p*=*0.040
Sex (% women)	2305 (55.8)	*d=*0.07, p*=*0.026	*d*=0.04, p*=*0.231
Total net financial wealth (£)		ɛ^2^=0.04, p*<*0.001	ɛ^2^=0.01, p*<*0.001
Quintile 1	603 (14.6)		
Quintile 2	638 (15.4)		
Quintile 3	861 (20.8)		
Quintile 4	977 (23.6)		
Quintile 5	1055 (25.5)		
Ethnicity (% white)	4094 (99.0)	*d=*0.08, p*=*0.642	*d=*0.01, p*=*0.894
Marital/cohabitation status (% married or cohabiting)	3023 (73.1)	*d*=0.29, p*<*0.001	*d*=0.29, p*<*0.001
Physical activity per week		ɛ^2^=0.03, p*<*0.001	ɛ^2^=0.01, p*<*0.001
Light or none	664 (16.1)		
Moderate or vigorous 1/week	1007 (24.4)		
Moderate or vigorous >1/week	2463 (59.6)		
Smoking (% smokers)	564 (13.6)	*d*=0.33, p*<*0.001	*d*=0.21, p*<*0.001
Alcohol consumption (% <5 days/week)	3097 (74.9)	*d*=0.13, p<0.001	*d*=0.09, p=0.029
BMI (kg/m^2^)	27.56±4.63	*r*=−0.03, p*=*0.043	*r*=0.01, p*=*0.409
Hypertension cases (% yes)	1698 (41.1)	*d*=0.11, p*<*0.001	*d*=0.01, p*=*0.735
CHD cases (% yes)	400 (9.7)	*d*=0.21, p*<*0.001	*d*=0.11, p=0.035
HbA1c† (mmol/mol)	36.2±2.46	*r*=−0.05, p*=*0.004	*r*=−0.04, p*=*0.007
Depression cases^a^ (% yes)	526 (12.7)	*d*=0.93, p*<*0.001	*d*=0.77, p*<*0.001

*Associations were tested with Pearson’s *r* correlations for continuous variables and independent samples t-tests and Kruskal-Wallis H tests for categorical variables.

†HbA1c (mmol/mol) levels equal to 5.46% ±0.44. *^a^n* =4104.

BMI, body mass index; CASP, Control Autonomy Self-realisation Pleasure scale; CHD, coronary heart disease; HbA1c, glycated haemoglobin; kg/m^2^, kilograms per square metre; mmol/mol, millimoles per mole; n, number; SD, standard deviation.

Three hundred and twelve incident cases of T2D were reported (7.5%) over the average 11.6-year follow-up period. Univariate analyses showed that those who developed T2D were more likely to have lower enjoyment of life scores at baseline compared to those who did not develop diabetes (p=0.001). In contrast, purpose in life scores did not differ between those who developed and did not develop T2D during the follow-up (p=0.143).

Cox regression analyses confirmed a significant inverse association between enjoyment of life and incident T2D in all five models. As shown in table 2, every unit increase in enjoyment of life was associated with an 11% reduction in the rate of T2D in unadjusted analysis (HR =0.89, p<0.001, 95% CI (0.84, 0.94)). Sociodemographic factors (age, sex, financial wealth, ethnicity, marital/cohabitation status) accounted for 27% of the association between enjoyment of life and T2D. Behavioural factors (physical activity, smoking status, alcohol consumption, BMI) accounted for 27% reduction in the HR for enjoyment of life compared with the unadjusted model. Clinical variables (hypertension, CHD, HbA1c) accounted for 18% of the association. In the final, fully adjusted model including all covariates, the significant association between enjoyment of life and T2D was maintained. Specifically, for every unit increase in reported enjoyment of life, there was a 7% reduction in the hazard of T2D (HR =0.93, p=0.020, 95% CI (0.87, 0.99)), with sociodemographic, behavioural and clinical factors combined accounting for a 36% reduction in the HR for enjoyment of life compared with the basic, unadjusted model.

**Table 2:** Cox proportional hazards regression on enjoyment of life at 2004/2005 predicting T2D incidence from 2006/2007 to 2016/2017—potential mediators and confounders (N=4134)

	HR	95% CI	P value	PPAE
Unadjusted model	0.89	0.84 to 0.94	<0.001	
+Sociodemographic variables	0.92	0.87 to 0.97	0.003	27%
+Behavioural variables	0.92	0.87 to 0.97	0.004	27%
+Clinical variables	0.91	0.86 to 0.97	0.003	18%
+Sociodemographic, behavioural, and clinical variables	0.93	0.87 to 0.99	0.020	36%

CI, confidence interval; HR, hazards ratio; N, number; PPAE, percentage of protective association explained; T2D, type 2 diabetes.

**Figure 2:**
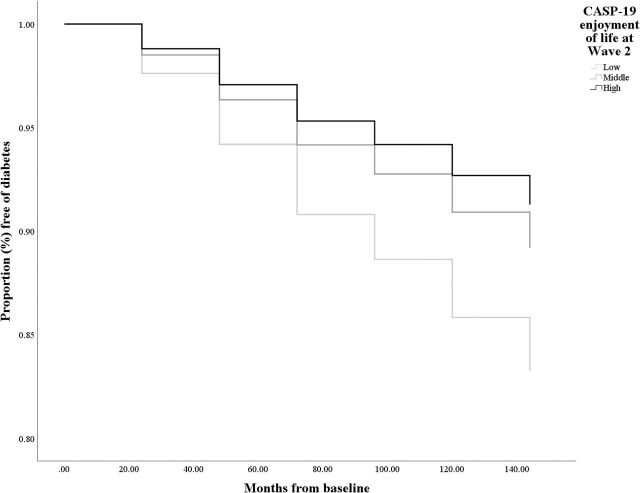
Kaplan-Meier survival curves for incident diabetes in low, middle and high enjoyment groups of participants in the English Longitudinal Study of Ageing cohort (N=4134). Horizontal axis=time in months since baseline (2004/2005). Results are adjusted for age and sex. CASP, Control Autonomy Self-realisation Pleasure scale.

The fully adjusted model is presented in table 3. In this model, enjoyment of life (HR =0.93, p=0.020, 95% CI (0.87 to 0.99)), sex (HR =0.64, p<0.001, 95% CI (0.50 to 0.81)), BMI (HR =1.10, p<0.001, 95% CI (1.08, 1.13)), hypertension (HR =1.35, p=0.013, 95% CI (1.07, 1.71)) and HbA1c (HR =2.58, p<0.001, 95% CI (2.33, 2.85)) were all significant predictors of T2D rate over the follow-up. Significant findings from the Cox regression analysis are also illustrated in figure 2. The full individual models of enjoyment of life are also presented in online supplemental tables S1–S3.

**Table 3:** Cox proportional hazards regression on enjoyment of life at 2004/2005 predicting T2D incidence from 2006/2007 to 2016/2017 after adjusting for sociodemographic, behavioural and clinical variables (N=4134)

	HR	95% CI	P value
CASP enjoyment of life score	0.93	0.87 to 0.99	0.020
Age (years)	1.01	1.00 to 1.03	0.070
Sex (reference cat.: men)	0.64	0.50 to 0.81	<0.001
Financial wealth (£)			
Quintile 1 (reference cat.)	1	0.67 to 1.52	0.962
Quintile 2	1.01	0.79 to 1.65	0.472
Quintile 3	1.14	0.67 to 1.42	0.891
Quintile 4	0.97	0.46 to 1.04	0.076
Quintile 5	0.69		
Ethnicity (reference cat.: white)	1.87	0.83 to 4.23	0.131
Marital/cohabiting status (reference cat.:married or cohabiting)	0.75	0.565 to 1.00	0.051
Physical activity per week			
Light or none (reference cat.)	1	0.66 to 1.34	0.737
Moderate or vigorous 1 per week	0.94	0.71 to 1.31	0.802
Moderate or vigorous >1 per week	0.96		
Smoking status (reference cat.: smoker)	0.94	0.68 to 1.30	0.708
Alcohol consumption (reference cat.: <5 days/week)	0.86	0.64 to 1.16	0.332
BMI (kg/m^2^)	1.10	1.08 to 1.13	<0.001
Hypertension (reference cat.: no)	1.35	1.07 to 1.71	0.013
CHD (reference cat.: no)	1.12	0.79 to 1.59	0.526
HbA1c (%)	2.58	2.33 to 2.85	<0.001

BMI, body mass index; HbA1c, glycated haemoglobin; HR, hazards ratio; kg/m^2^, kilograms per square metre; N, number; cat, category; T2D, type 2 diabetes.

10.1136/jech-2020-214302.supp1 Supplementary data

Purpose in life showed a significant inverse association with T2D in unadjusted analysis. More specifically, for every unit increase in purpose in life, there was a 15% reduction in the hazard of T2D (HR =0.85, p=0.032, 95% CI (0.74, 0.99)). As shown in table 4, the association between purpose in life and T2D was attenuated in models adjusted for sociodemographic variables (HR =0.90, p=0.154, 95% CI (0.77, 1.04)), behavioural variables (HR =0.87, p=0.066, 95% CI (0.75, 1.01)), clinical variables (HR =0.93, p=0.389, 95% CI (0.80, 1.09)) and in the fully adjusted model (HR =0.92, p=0.322 95% CI (0.79, 1.08)). Compared with the unadjusted model, sociodemographic variables together accounted for 33% of the association between purpose in life and T2D, behavioural factors accounted for 13% of the association and clinical factors for 53%. Sociodemographic, behavioural and clinical factors combined accounted for a 47% reduction in the HR for purpose in life compared with the basic, unadjusted model. The full individual models of purpose in life are presented in online supplemental tables S4–S7.

**Table 4:** Cox proportional hazards regression on purpose in life at 2004/2005 predicting T2D incidence from 2006/2007 to 2016/2017—potential mediators and confounders (N=4134)

	HR	95% CI	P value	PPAE
Unadjusted model	0.85	0.74 to 0.99	0.032	
+Sociodemographic variables	0.90	0.77 to 1.04	0.154	33%
+Behavioural variables	0.87	0.75 to 1.01	0.066	13%
+Clinical variables	0.93	0.80 to 1.09	0.389	53%
+Sociodemographic, behavioural and clinical variables	0.92	0.79 to 1.08	0.322	47%

CI, confidence interval; HR, hazards ratio, n, number; PPAE, percentage of protective association explained; T2D, type 2 diabetes.

Depression was included in a secondary model along with enjoyment of life, age and sex to test for an independent association between enjoyment of life and T2D rate. Results revealed a significant role for enjoyment of life independently of depression (HR =0.91, p=0.002, 95% CI (0.86, 0.97); see online supplemental table S8). The association between purpose in life and T2D adjusted for age, sex and depression was not significant (HR =0.91, p=0.222, 95% CI (0.78, 1.06)). Secondary analyses also indicated that the relationship between the two exposure variables, enjoyment of life and purpose in life and T2D incidence did not differ according to age (age by enjoyment of life: fully adjusted HR =1.00, p=0.287, 95% CI (1.00 to 1.01); age by purpose in life: fully adjusted HR =1.01, p=0.556, 95% CI (0.99, 1.02)) or sex (sex by enjoyment of life: fully adjusted HR =0.96, p=0.477, 95% CI (0.85, 1.08); sex by purpose in life: fully adjusted HR =0.88, p=0.435, 95% CI (0.65, 1.21)).

In our first sensitivity analysis, 70 participants who developed T2D within two years from baseline (by Wave 3; 2006/2007) were excluded (n=4064) and the association between enjoyment of life and T2D remained significant (fully adjusted HR =0.92, p=0.021, 95% CI (0.86, 0.99)). Second, the main analysis was repeated after excluding possible undiagnosed cases of T2D at baseline. Sixty-six participants with HbA1c value ≥48 mmol/mol (equals to ≥6.5%) at baseline were excluded (n=4068) and the association between enjoyment of life and T2D remained significant (fully adjusted HR =0.93, p=0.029, 95% CI (0.87, 0.99)). Finally, purpose in life analyses were repeated using a binary measure instead of a continuous variable. The relationship between purpose in life and incident T2D was not significant in either unadjusted (HR =0.74, p=0.111, 95% CI (0.51, 1.07)) or fully adjusted analysis (HR =0.75, p=0.135, 95% CI (0.51, 1.09)).

## DISCUSSION

We investigated the longitudinal association between two different components of subjective well-being and incident T2D over a period of 12 years. We found evidence of a protective relationship between enjoyment of life and rate of diabetes onset. Specifically, a 1-unit increase in enjoyment of life was associated with 7% reduction in the hazard of T2D. These findings were robust to adjustment for a range of covariates. Moreover, results revealed that the link between enjoyment of life and T2D could, in part, be attributed to sociodemographic, behavioural and clinical factors. The significant, inverse relationship between enjoyment and T2D was upheld after the exclusion of participants who developed T2D within two years from baseline and after the exclusion of participants with undiagnosed, objectively measured T2D at baseline.

The relationship between purpose in life and reduced T2D rate was significant in unadjusted analysis, but it was attenuated in models adjusting for covariates. This finding might suggest a non-direct effect of purpose in life in T2D and warrants investigation in future research. However, the use of a single purpose in life rating may have played a role in the null results. A previous meta-analysis showed that the purpose in life—physical health relationship is stronger when measures combine items referring to meaning in life and meaning-related sense of harmony, peace and well-being, compared with items focusing solely on meaning in life.^31^ Nevertheless, a single question has been applied in previous studies of older adults, showing significant, inverse associations with number of chronic illnesses.^2 32^ Overall, our results support the idea that T2D is differentially related to hedonic and eudaimonic well-being, adding value to testing the different dimensions separately.

The maintenance of the significant association between enjoyment and T2D in secondary analysis adjusting for depression provides further evidence of the direct, independent association between hedonic well-being and T2D, in line with previous studies,^5 19^ and lends support to the notion that hedonic well-being is not always secondary to the absence of psychological distress or negative affect.^9^ Secondary analyses also tested for potential age or sex differences in the links between enjoyment and T2D and purpose and T2D. Similar results emerged across younger and older adults and for both sexes. These findings contradict the age- and sex-dependent results described in previous studies^5 19 20^ and provide evidence for a more general, protective role for enjoyment of life. Inconsistent findings might be influenced by the precise measures of well-being, sample sizes^5 20^ or cultural differences.^20^

Sociodemographic, behavioural and clinical factors did not fully explain the protective association between enjoyment of life and T2D. Future studies need to investigate the role of additional mechanisms linking hedonic well-being with T2D. For example, enjoyment of life may have an impact on biological processes relevant to T2D, modulated via corticolimbic pathways. We have previously found that hedonic well-being is associated with reduced cortisol output over the day^33^ and lower inflammatory levels.^34^ In turn, dysregulated diurnal cortisol output and elevated inflammatory factors have been prospectively linked to T2D risk.^35 36^ Laboratory studies have also shown that hedonic well-being is associated with reduced inflammatory and cardiovascular reactivity, establishing a dynamic association between hedonic well-being and stress-related biological.^37^

Our study has several strengths. We included a large sample of participants derived from a nationally representative cohort. Models were differentiated between types of well-being and a series of analyses allowed us to estimate the proportion of association explained by the different covariates. BMI, blood pressure and HbA1c were assessed objectively during the nurse visit. The longitudinal design of the study enabled the examination of T2D incidence using a relatively long follow-up period. Additionally, the reverse causality argument was ruled out by excluding individuals with objective T2D at baseline and those who developed T2D within two years from baseline. Nevertheless, this study is not without limitations. Subjective well-being was assessed at a single time point; therefore, changes over time in these measures were not considered. Nevertheless, the temporal stability of subjective well-being has been previously documented.^38^ Patient reports of T2D diagnosis were used instead of objective clinical records, but a high agreement between self-reported and clinically derived diagnoses of T2D has been reported.^39^ Although multiple covariates were taken into account, we did not consider other potential covariates such as diet.^40^ Finally, the number of ethnic minority participants in ELSA is small; thus, our results may not generalise to non-white individuals.

In conclusion, this study provides evidence for the health-protective relationship between enjoyment in life and T2D incidence. Associations were only partially explained by sociodemographic, behavioural and clinical risk factors. One implication is that efforts to increase enjoyment of life in middle- and older-aged adults might help delay the onset of T2D, though further research is required to test this hypothesis.

**What is already known on this subject**

- Subjective well-being appears to be associated with lower risk of future type 2 diabetes (T2D). However, no previous studies examined separate associations between hedonic and eudaimonic well-being and T2D risk. Also, the proportion of association explained by sociodemographic, behavioural and clinical characteristics is still unknown.

**What does this study add**

- Hedonic well-being but not eudaimonic well-being is associated with reduced risk of T2D. This relationship is partially explained by sociodemographic and behavioural factors, and to a lesser extent, clinical characteristics.
