# Supplementary material for: Enjoyment of life predicts reduced type 2 diabetes incidence over 12 years of follow-up: findings from the English Longitudinal Study of Ageing
Source: J Epidemiol Community Health. 2020 Oct 21;75(3):297–304. doi: 10.1136/jech-2020-214302 (PMC7892367; doi:10.1136/jech-2020-214302)
Supplement: Supplementary data [file jech-2020-214302supp001.pdf]

Table S1

Cox proportional hazards regression on enjoyment of life at 2004/5 predicting T2D incidence from 2006/7 to 2016/7 after adjustment for sociodemographic variables (N = 4134)

	HR	95% CI	*p* value
CASP enjoyment of life score	0.916	0.865 to 0.971	0.003
Age [years]	1.013	0.999 to 1.027	0.060
Sex [reference cat.: men]	0.607	0.482 to 0.765	<0.001
Financial wealth (£)			
Quintile 1 [reference cat.]	1		
Quintile 2	0.593	0.407 to 0.865	0.007
Quintile 3	0.659	0.473 to 0.918	0.014
Quintile 4	0.521	0.372 to 0.729	<0.001
Quintile 5	0.332	0.229 to 0.481	<0.001
Ethnicity [reference cat.: white]	2.045	0.910 to 4.595	0.083
Marital/cohabiting status [reference cat.: married or cohabiting]	0.872	0.661 to 1.150	0.331

*Not*e. CASP = Control Autonomy Self-realisation Pleasure scale; CI = confidence interval; HR = hazard ratio; N = number; cat. = category; T2D = type 2 diabetes.

Table S2

Cox proportional hazards regression on enjoyment of life at 2004/5 predicting T2D incidence from 2006/7 to 2016/7 after adjusting for behavioural variables (N = 4134)

	HR	95% CI	*p* value
CASP enjoyment of life score	0.917	0.865 to 0.973	0.004
Physical activity per week			
Light or none [reference cat.]	1		
Moderate or vigorous 1 per week	0.731	0.523 to 1.022	0.067
Moderate or vigorous >1 per week	0.766	0.575 to 1.022	0.070
Smoking status [reference cat.: smoker]	0.668	0.497 to 0.898	0.007
Alcohol consumption [reference cat.: < 5 days/week]	0.799	0.597 to 1.069	0.131
BMI [kg/m^2]	1.109	1.089 to 1.130	<0.001

*Not*e. CASP = Control Autonomy Self-realisation Pleasure scale; CI = confidence interval; HR = hazard ratio; N = number; cat. = category; T2D = type 2 diabetes.

Table S3

Cox proportional hazards regression on enjoyment of life at 2004/5 predicting T2D incidence from 2006/7 to 2016/7 after adjustment for clinical variables (N = 4134)

	HR	95% CI	*p* value
CASP enjoyment of life score	0.913	0.860 to 0.970	0.003
Hypertension [reference cat.: no]	1.654	1.313 to 2.082	<0.001
CHD [reference cat.: no]	1.387	0.988 to 1.947	0.059
HbA1c [%]	2.735	2.519 to 2.970	<0.001

Note. CASP = Control Autonomy Self-realisation Pleasure scale; CI = confidence interval; CHD = coronary heart disease; HbA1c = glycated haemoglobin; HR = hazard ratio; N = number; cat. = category; T2D = type 2 diabetes.

Table S4

Cox proportional hazards regression on purpose in life at 2004/5 predicting T2D incidence from 2006/7 to 2016/7 after adjustment for sociodemographic variables (N = 4134)

	HR	95% CI	*p* value
CASP purpose in life score	0.898	0.774 to 1.041	0.154
Age [years]	1.013	1.000 to 1.027	0.057
Sex [reference cat.: men]	0.598	0.475 to 0.754	<0.001
Financial wealth (£)			
Quintile 1 [reference cat.]	1		
Quintile 2	0.581	0.339 to 0.846	0.005
Quintile 3	0.630	0.453 to 0.887	0.006
Quintile 4	0.499	0.357 to 0.697	<0.001
Quintile 5	0.313	0.217 to 0.452	<0.001
Ethnicity [reference cat.: white]	2.031	0.903 to 4.564	0.086
Marital/cohabiting status [reference cat.: married or cohabiting]	0.886	0.671 to 1.169	0.391

Note. CASP = Control Autonomy Self-realisation Pleasure scale; CI = confidence interval; HR = hazard ratio; N = number; cat. = category; T2D = type 2 diabetes.

Table S5

Cox proportional hazards regression on purpose in life at 2004/5 predicting T2D incidence from 2006/7 to 2016/7 after adjusting for behavioural variables (N = 4134)

	HR	95% CI	*p* value
CASP purpose in life score	0.869	0.749 to 1.009	0.066
Physical activity per week			
Light or none [reference cat.]	1		
Moderate or vigorous 1 per week	0.712	0.509 to 0.996	0.047
Moderate or vigorous >1 per week	0.741	0.556 to 0.986	0.040
Smoking status [reference cat.: smoker]	0.646	0.482 to 0.867	0.004
Alcohol consumption [reference cat.: < 5 days/week]	0.789	0.590 to 1.056	0.111
BMI [kg/m^2]	1.110	1.090 to 1.131	<0.001

*Not*e. BMI = body mass index; CASP = Control Autonomy Self-realisation Pleasure scale; CI = confidence interval; HR = hazard ratio; kg/m^2 = kilograms per square metre; N = number; cat. = category; T2D = type 2 diabetes.

Table S6

Cox proportional hazards regression on purpose in life at 2004/5 predicting T2D incidence from 2006/7 to 2016/7 after adjusting for clinical variables (N = 4134)

	HR	95% CI	*p* value
CASP purpose in life score	0.933	0.797 to 1.092	0.389
Hypertension [reference cat.: no]	1.685	1.339 to 2.121	<0.001
CHD [reference cat.: no]	1.411	1.006 to 1.981	0.046
HbA1c [%]	2.751	2.531 to 2.990	<0.001

*Not*e. CASP = Control Autonomy Self-realisation Pleasure scale; CHD = coronary heart disease; CI = confidence interval; HbA1c = glycated haemoglobin; HR = hazard ratio; N = number; cat. = category; T2D = type 2 diabetes.

Table S7

Cox proportional hazards regression on purpose in life at 2004/5 predicting T2D incidence from 2006/7 to 2016/7 after adjusting for sociodemographic, behavioural, and clinical variables (N = 4134)

	HR	95% CI	*p* value
CASP purpose in life score	0.923	0.787 to 1.082	0.322
Age [years]	1.014	0.999 to 1.030	0.064
Sex [reference cat.: men]	0.632	0.497 to 0.804	<0.001
Financial wealth (£)			
Quintile 1 [reference cat.]	1		
Quintile 2	0.998	0.664 to 1.499	0.993
Quintile 3	1.115	0.775 to 1.605	0.556
Quintile 4	0.995	0.657 to 1.388	0.810
Quintile 5	0.669	0.443 to 1.012	0.057
Ethnicity [reference cat.: white]	1.865	0.826 to 4.213	0.134
Marital/cohabiting status [reference cat.: married or cohabiting]	0.760	0.571 to 1.013	0.061
Physical activity per week			
Light or none [reference cat.]	1		
Moderate or vigorous 1 per week	0.932	0.654 to 1.328	0.697
Moderate or vigorous >1 per week	0.942	0.694 to 1.279	0.701
Smoking status [reference cat.: smoker]	0.916	0.662 to 1.266	0.594
Alcohol consumption [reference cat.: < 5 days/week]	0.846	0.628 to 1.141	0.273
BMI [kg/m^2]	1.105	1.082 to 1.129	<0.001
Hypertension [reference cat.: no]	1.365	1.077 to 1.729	0.010
CHD [reference cat.: no]	1.124	0.793 to 1.593	0.512
HbA1c [%]	2.558	2.314 to 2.827	<0.001

*Not*e. BMI = body mass index; CASP = Control Autonomy Self-realisation Pleasure scale; CHD = coronary heart disease; CI = confidence interval; HbA1c = glycated haemoglobin; HR = hazard ratio; kg/m^2 = kilograms per square metre; N = number; cat. = category; T2D = type 2 diabetes.

Table S8

Cox proportional hazards regression on enjoyment of life at 2004/5 predicting T2D incidence from 2006/7 to 2016/7 after adjusting for covariates including depressive symptoms (N = 4134)

	HR	95% CI	*p* value
CASP enjoyment of life score	0.910	0.857 to 0.967	0.002
Age [years]	1.009	0.996 to 1.023	0.163
Sex [reference cat.: men]	0.599	0.477 to 0.752	<0.001
Depressive symptoms score	1.422	1.030 to 1.963	0.033

*Not*e. CASP = Control Autonomy Self-realisation Pleasure scale; CI = confidence interval; HR = hazard ratio; N = number; T2D = type 2 diabetes.
